# SARS-CoV-2 engages inflammasome and pyroptosis in human primary monocytes

**DOI:** 10.1038/s41420-021-00428-w

**Published:** 2021-03-01

**Authors:** André C. Ferreira, Vinicius Cardoso Soares, Isaclaudia G. de Azevedo-Quintanilha, Suelen da Silva Gomes Dias, Natalia Fintelman-Rodrigues, Carolina Q. Sacramento, Mayara Mattos, Caroline S. de Freitas, Jairo R. Temerozo, Lívia Teixeira, Eugenio Damaceno Hottz, Ester A. Barreto, Camila R. R. Pão, Lohanna Palhinha, Milene Miranda, Dumith Chequer Bou-Habib, Fernando A. Bozza, Patrícia T. Bozza, Thiago Moreno L. Souza

**Affiliations:** 1 Laboratório de Imunofarmacologia, Instituto Oswaldo Cruz (IOC), Fundação Oswaldo Cruz (Fiocruz), Rio de Janeiro, RJ Brazil; 2 Laboratório de Pesquisa Pré-clínica—Universidade Iguaçu - UNIG, Nova Iguaçu, RJ Brazil; 3 National Institute for Science and Technology on Innovation in Diseases of Neglected Populations (INCT/IDPN), Center for Technological Development in Health (CDTS), Fiocruz, Rio de Janeiro, RJ Brazil; 4 Program of Immunology and Inflammation, Federal University of Rio de Janeiro, UFRJ, Rio de Janeiro, RJ Brazil; 5 Laboratório de Pesquisas sobre o Timo, IOC, Fiocruz, Rio de Janeiro, RJ Brazil; 6 National Institute for Science and Technology on Neuroimmunomodulation, Oswaldo Cruz Institute, Fiocruz, Rio de Janeiro, RJ Brazil; 7 Laboratório de Imunotrombose, Departamento de Bioquímica, Universidade Federal de Juiz de Fora, Juiz de Fora, MG Brazil; 8 Laboratório de Vírus Respiratório e do Sarampo, IOC, Fiocruz, Rio de Janeiro, RJ Brazil; 9 Instituto Nacional de Infectologia Evandro Chagas, Fiocruz, Rio de Janeiro, RJ Brazil; 10 Instituto D’or de Pesquisa e Ensino, Rio de Janeiro, RJ Brazil

**Keywords:** Viral infection, Inflammasome

## Abstract

Infection by the severe acute respiratory syndrome coronavirus 2 (SARS-CoV-2) has been associated with leukopenia and uncontrolled inflammatory response in critically ill patients. A better comprehension of SARS-CoV-2-induced monocyte death is essential for the identification of therapies capable to control the hyper-inflammation and reduce viral replication in patients with 2019 coronavirus disease (COVID-19). Here, we show that SARS-CoV-2 engages inflammasome and triggers pyroptosis in human monocytes, experimentally infected, and from patients under intensive care. Pyroptosis associated with caspase-1 activation, IL-1ß production, gasdermin D cleavage, and enhanced pro-inflammatory cytokine levels in human primary monocytes. At least in part, our results originally describe mechanisms by which monocytes, a central cellular component recruited from peripheral blood to respiratory tract, succumb to control severe COVID-19.

## Introduction

Severe acute respiratory coronavirus 2 (SARS-CoV-2), the etiological agent of the 2019 coronavirus disease (COVID-19), emerged in China, causing a major public health burden in decades. Although patients with severe COVID-19 may present an asymptomatic/mild disease, others experience acute respiratory distress syndrome (ARDS) characterized by elevated serum levels of proinflammatory mediators—the cytokine storm^1–3^. Patients with severe COVID-19, monocytes/macrophages may be the main source of uncontrolled levels of the pro-inflammatory mediators TNF-α and IL-6 in the respiratory tract in peripheral blood^4^. Plasmatic levels of IL-6 have been associated with mortality, intensive care admission and hospitalization, representing a poor prognostic factor for COVID-19^5^. The uncontrolled inflammation triggered by SARS-CoV-2 occurs 7 to 10 days after onset of illness and associates with decreased viral loads^6,7^.

In severe COVID-19, the cytokine storm associates with high levels of tissue insult, judged by increased levels lactate dehydrogenase (LDH) and D-dimer in the plasma^6–8^. Moreover, high LDH levels and leukopenia in severe COVID-19 points out that white cells loses the integrity of plasma membrane^8–11^. Among these cells, monocytes should orchestrate the equilibrium between innate and adaptative immune responses, which may be presumably affected during cytokine storm. The leukopenia of patients with severe COVID-19 seem to precede the cytokine storm^11,12^. Moreover, in other virus-induced cytokine storm episodes in the respiratory tract, such as induced by influenza A virus, monocytes and macrophages are severely affected^13–16^.

There are various mechanisms involved in lytic cell death, which are differently engaged from development to responses to infection^17^. For certain diseases, such as COVID-19, in which the immunopathogenesis mechanisms associate with poor clinical outcomes, controlling the way cells collapse to infection is vital for the host^17^. Lytic cell death may be triggered by pyroptosis or necroptosis, in monocytes/macrophages this phenomenon exacerbates inflammation, because of release of intracellular damage-associated molecular patterns^18,19^. Pyroptosis is a caspase-1-dependent event that leads to gasdermin D (GSDMD) pore formation^20^. Necroptosis depends on intracellular signaling of receptor-interacting protein kinases (RIPK) and mixed lineage kinase domain-like phosphorylation to form membrane pores^20^. Thus, we hypothesized that the monocyte cell death induced by SARS-COV-2 exacerbates the production of inflammatory cytokines, as well as impairs the immune balance in the hosts. In fact, we found that SARS-CoV-2 engages inflammasomes and pyroptosis in human monocytes, by experimental or natural infection. These events were associated with caspase-1 activation, IL-1ß production, GSDMD cleavage and dysregulation of cytokine release. Finally, we show that inhibition of early steps of SARS-CoV-2 life cycle by atazanavir (ATV) could block pyroptosis in SARS-CoV-2-infected human primary monocytes.

## Results

### Lytic cell death in SARS-CoV-2-infected human primary monocytes

**Fig. 1:**
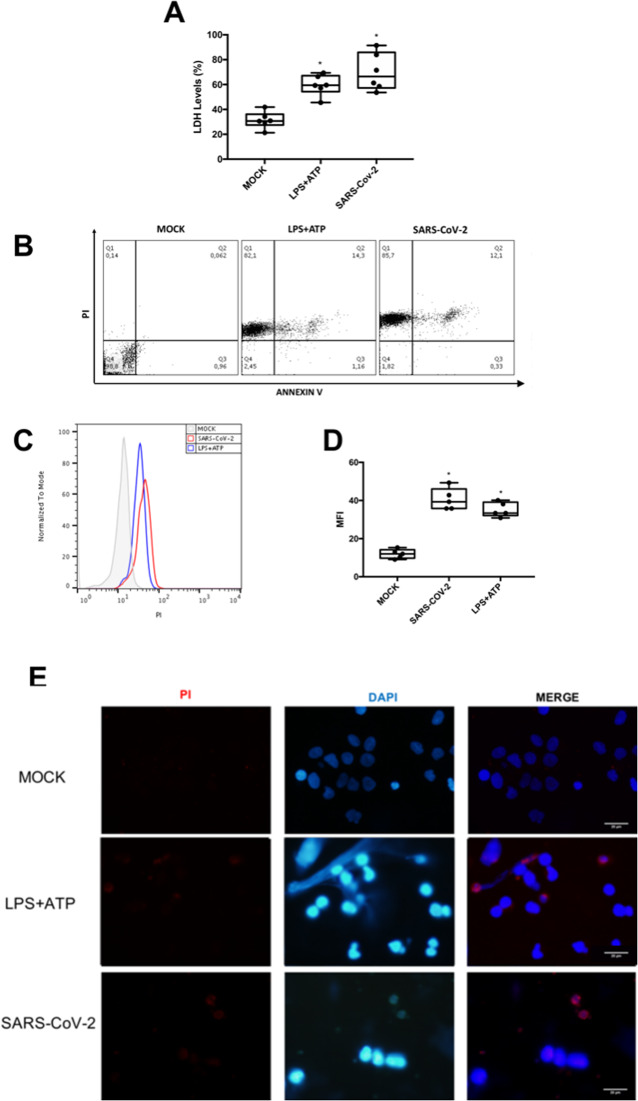
SARS-CoV-2 induces lytic monocyte cell death. Human monocytes were infected with SARS-CoV-2 (MOI 0.1) for 24 h. As a positive control, monocytes were stimulated with LPS (500 ng/mL) for 23 h and, after this time, incubated with ATP (2 mM) for 1 h. **A** Cell viability was assessed through the measurement of LDH levels in the culture supernatant. **B**–**D** Monocytes were stained with propidium iodide (PI) and Annexin V. **E** Monocytes were stained with PI and DAPI, scale bar 20 μm. Images and graph data are representative of six independent experiments. Data are presented as the mean ± SEM *P < 0.05 versus control group (MOCK-infected).

For initial assessments of SARS-CoV-2-induced monocyte death, we quantified the LDH activity in the culture supernatant and annexinV/propidium iodide (PI) labeling through flow cytometry and fluorescence microscopy. SARS-CoV-2 increased LDH levels similarly to the positive control, LPS + ATP (Fig. 1A). SARS-CoV-2 was as potent as LPS + ATP to increase PI^+^ cells by 300-fold and PI^+^/annexinV^+^ cells by 200-fold (Fig. 1B). Flow cytometry and fluorescence microscopy also reconfirm plasma membrane disruption triggered by SARS-CoV-2 in a similar magnitude to the positive control (Fig. 1B–E). These data suggest that SARS-CoV-2 infection can induce lytic cell death in human primary monocytes.

### Inflammasome engagement and pyroptotic events in SARS-CoV-2-infected monocytes

**Fig. 2:**
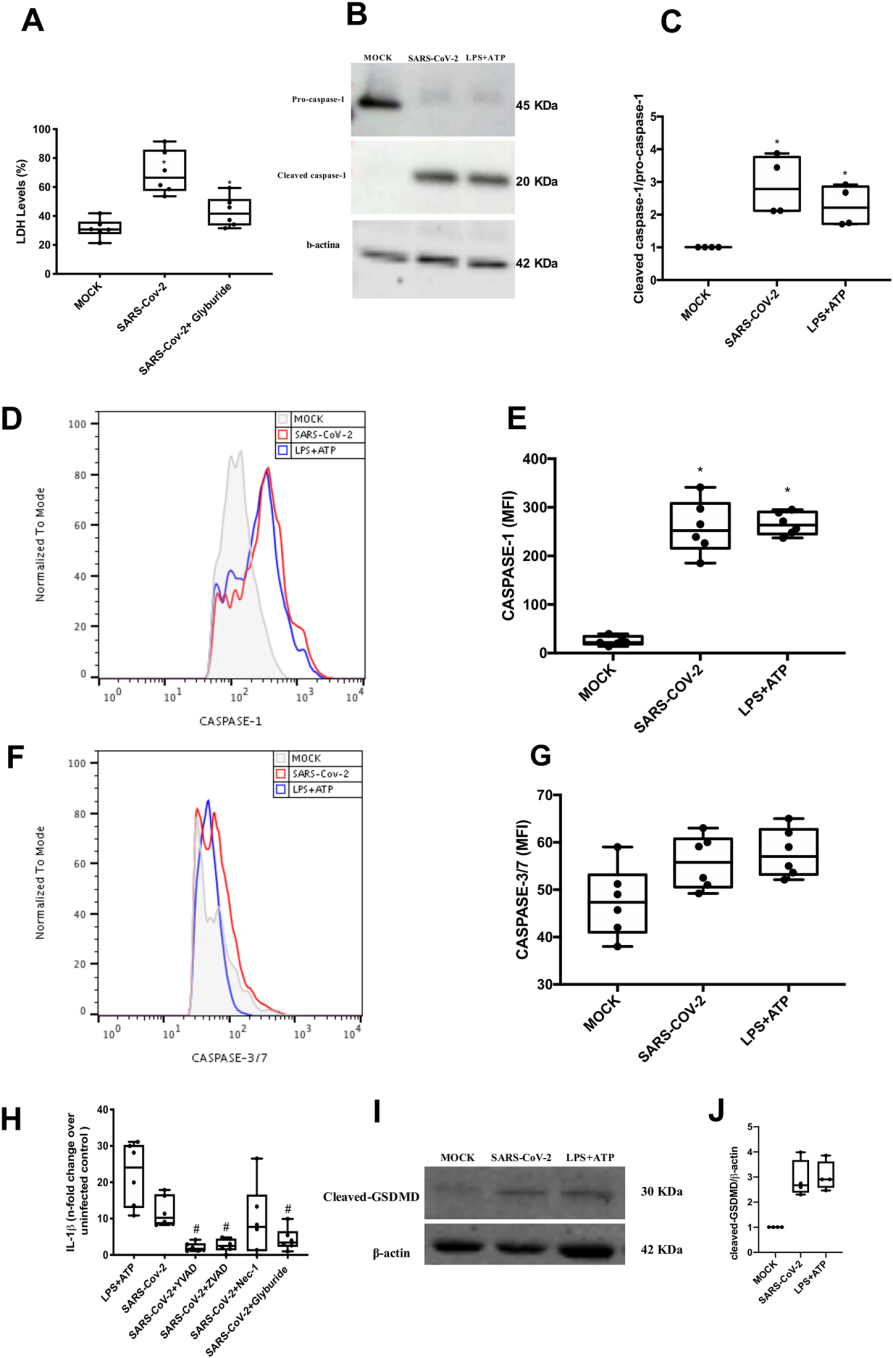
Lytic cell death in SARS-CoV-2-infected monocytes associates with inflammasome engagement and pyroptosis. Human monocytes were treated with pharmacological inhibitors to impair the function of the following proteins: NLPR3 (Gliburyde; 100 µM), caspase-1 (AC-YVAD-CMK; 1 µM), or pan-caspase (Z-VAD-FMK; 10 µM) or RIPK (Nec-1; 25 µM). Monocytes were treated since 1 h prior to infection with SARS-CoV-2 (MOI 0.1), for 24 h. As a control, monocytes were stimulated with LPS (500 ng/mL) for 23 h and, after this time, stimulated with ATP (2 mM) for 1 h. **A** Cell viability was assessed through the measurement of LDH levels in the supernatant of monocytes. **B**, **C** Monocytes were lysed and used for determination of pro-caspase-1 and cleaved caspase-1 levels by western blotting. **D**, **E** Monocytes were stained with FAM-YVAD-FLICA to determine the caspase-1 activity by flow cytometry. **F**, **G** Monocytes were stained with FAM-FLICA to determine caspase-3/7 activity by flow cytometry. **H** Cell culture supernatants were collected for the measurement of the levels of IL-1β. **I**, **J** Monocytes were lysed and cleaved GSDMD levels were determined western blotting. Western blotting images, histogram and graph data are representative of six independent experiments. Data are presented as the mean ± SEM ^#^*P* < 0.05 versus infected and untreated group; ^*^P < 0.05 versus control group (MOCK-infected).

Severe COVID-19 was associated with inflammasome activation^21^. Indeed, we observed that glyburide, which prevents NLR family pyrin domain containing 3 (NLRP3) activation^22^, abolished SARS-CoV-2-dependent monocyte lytic death (Fig. 2A). Next, we measured caspase-1 activation, a downstream event to inflammasome engagement^20^. SARS-CoV-2 induced pro-caspase-1 cleavage, similarly to LPS + ATP, by western blotting and flow cytometry analysis (Fig. 2B, C). Apoptotic caspases-3/-7 were not upregulated in SARS-CoV-2-infected monocytes (Fig. 2F, G).

Caspase-1 activation is known to trigger IL-1β production and GSDMD cleavage^20–24^. Indeed, SARS-CoV-2 infection increased IL-1β production, which was prevented by selective inhibition of capase-1 (AC-YVAD-CMK) or by treatment with pan-caspase inhibitor (Z-VAD-FMK) (Fig. 2H). Impairment of NLPR3, by glyburide, also prevented SARS-CoV-2-induced IL-1β production (Fig. 2H). RIPK inhibition by necrostatin could not prevent significantly IL-1β production (Fig. 2H).

Based on the pharmacological characterization, SARS-CoV-2 depends on cellular components associated with pyroposis to promote lytic monocyte death. We thus measured the cleaved GSDMD as a proxy of the pore forming structure. In fact, SARS-CoV-2, similarly to LPS + ATP, enhanced cleaved GSDMD levels (Fig. 2I). Importantly, inhibition of IL-1R engagement reduced SARS-CoV-2-mediated caspase-1 activation and cell lysis (Fig. S1A, B), suggesting that inflammasome-dependent IL-1β secretion amplify caspase-1 activation and pyroptosis in SARS-CoV-2 infection.

### Inflammasome activation amplify pro-inflammatory cytokines secretion in infected monocytes

**Fig. 3:**
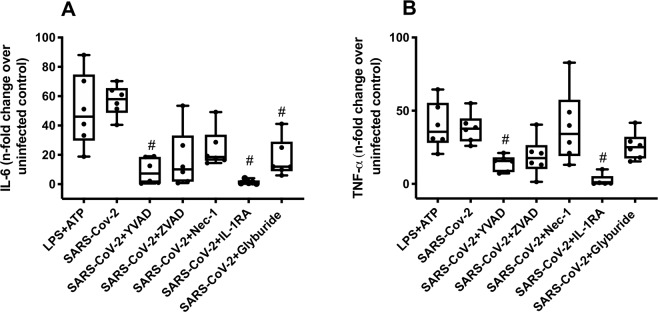
Caspase-1- and IL-1 receptor-dependent amplification pro-inflammatory cytokines secretion in SARS-CoV-2-infected monocytes. Human monocytes were pharmacologically treated to impair the function of caspase-1 (AC-YVAD-CMK; 1 µM), pan-caspase (ZVAD-FMK; 10 µM), RIPK (Nec-1; 25 µM), IL-1ß receptor (IL-1RA; 1 µM), or NLPR3 (glyburide 100 µM) since 1 h prior to infection at MOI of 0.1 with SARS-CoV-2. Monocytes were stimulated with LPS (500 ng/mL) for 23 h and after this time were stimulated with ATP (2 mM) for 1 h as a positive control. Cell culture supernatants were collected for the measurement of the levels of **A** IL-6 and **B** TNF-α. Graphs data are representative of six independent experiments. Data are presented as the mean ± SEM ^#^*P* < 0.05 versus infected and untreated group.

We subsequently quantified the levels of IL-6 and TNF-α, in SARS-CoV-2-infected monocytes upon pharmacological treatment to block inflammasome and pyroptosis. Inhibition of caspase-1, NLP3, and IL-1 receptor prevented SARS-CoV-2-induced enhancement of IL-6 and TNF-α levels (Fig. 3A, B). In addition, inhibition of IL-1 receptor engagement also reduced SARS-CoV-2-dependent production of IL-1 β (Fig. S1C). Moreover, IL-8 production, which is independent of inflammasome engagement and pyroptosis, was only modulated by pan-caspase inhibition in SARS-CoV-2-infeced monocytes (Fig. S2).

### ATV prevented SARS-CoV-2-induced inflammasome-engagement and pyroptosis

**Fig. 4:**
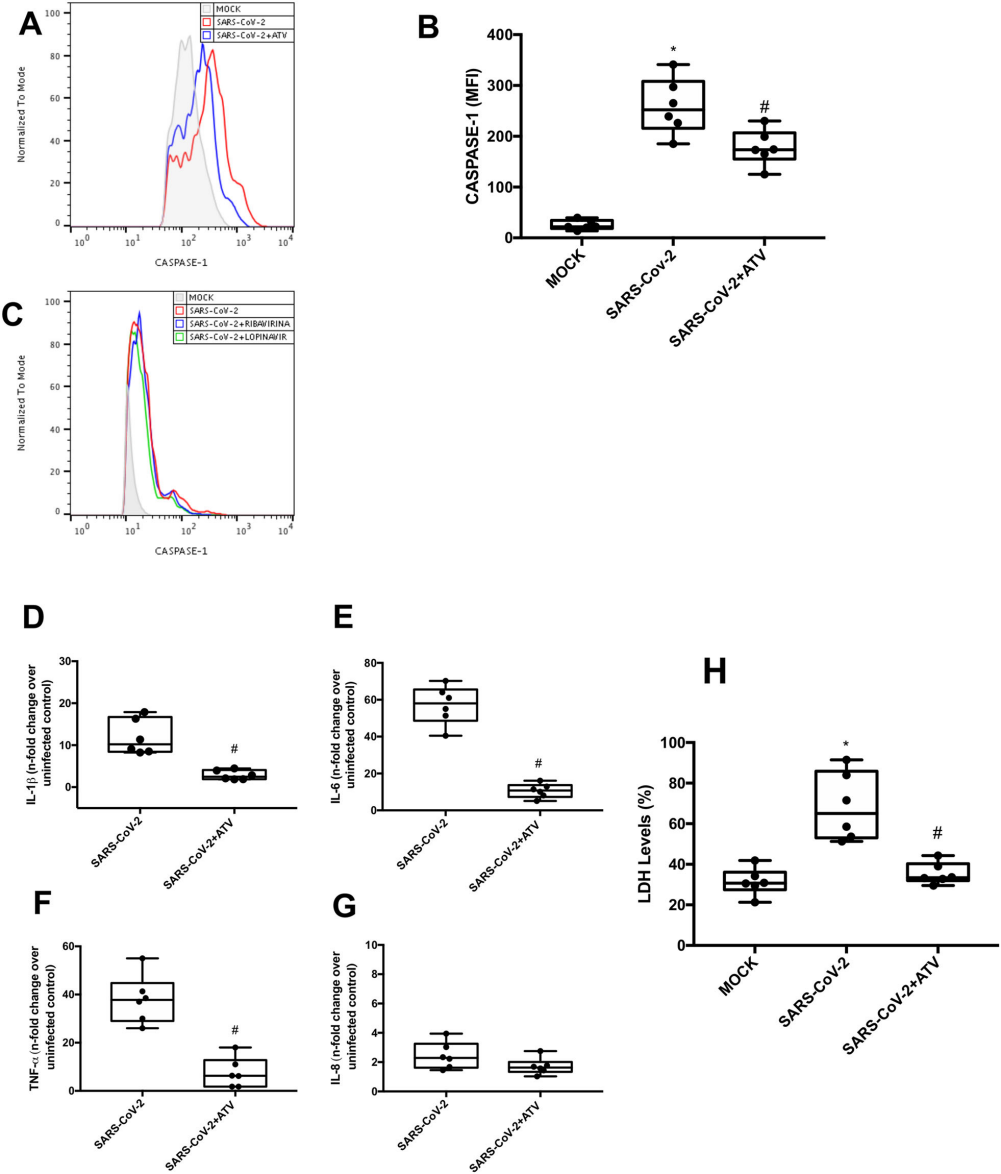
Atazanavir prevented capase-1-dependent lytic monocyte death. Human monocytes were infected with SARS-CoV-2 and treated with atazanavir (ATV; 10 µM), ribavirina (10 µM), or Lopinavir (10 µM) for 24 h. **A**, **B** Monocytes were stained with FAM-YVAD-FLICA to determine caspase-1 activity. **C** Monocytes were stained with FAM-FLICA to determine caspase-3/7 activity. **D**–**G** Culture supernatants were collected and the levels of IL-1β, IL-6, TNF-α, and IL-8 were determined by ELISA. **H** Assessment of cell viability through the measurement of LDH levels in the supernatant of monocytes. Histogram and graphs data are representative of six independent experiments. Data are presented as the mean ± SEM ^*^ *P* < 0.05 versus control group (MOCK-infected); ^#^*P* < 0.05 versus only infected group.

Since lytic cell death in monocytes may depend on SARS-CoV-2 replication, we tested whether orally available generic antivirals could prevent these events. Flow cytometry analysis of SARS-CoV-2-infected monocytes treated with ATV demonstrated a significant reduction in caspase-1 activity (Fig. 4A, B). Other orally available repurposed anti-SARS-CoV-2 drugs, such as lopinavir (LPV) and ribavirin (RBV), did not affect SARS-CoV-2-induced caspase-1 activation (Fig. 4C). Moreover, treatments with ATV did not alter the activity of caspase-1, -3 and -7 in monocytes exposed to LPS + ATP (Fig. S3), indicating a specific antiviral activity of this drug. Consistently, treatment with ATV also reduced the levels of IL-1β, IL-6, and TNF-α in SARS-CoV-2-infected monocytes, when compared to the untreated cells (Fig. 4D–F). ATV did not interfere with the production of IL-8, which is independent of virus-induced inflammasome engagement (Fig. 4G). Consistently, lower levels of LDH were detected in the supernatants of SARS-CoV-2-infected monocytes treated with ATV, when compared to infected and untreated cells (Fig. 4H). Since ATV inhibits the early proteolytic processing of viral antigens, an early event during SARS-CoV-2 replication, this drug represented the most upstream process pharmacologically inhibited in this investigation to prevent lytic monocyte death.

### Caspase 1- activation, lytic monocyte death, and Il-1β associate with severe COVID-19

**Fig. 5:**
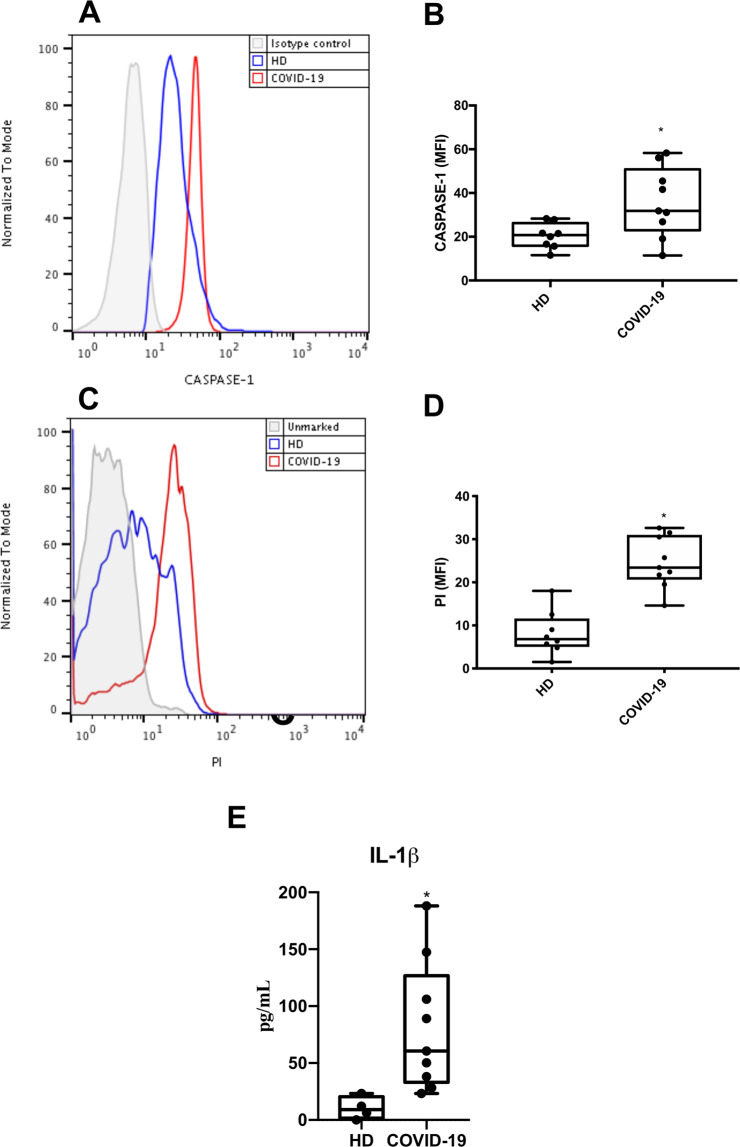
Caspase 1 activation, lytic monocyte death, and increased Il-1β lvels associate with severe COVID-19. Monocytes were isolated from blood samples of critically ill patients with COVID-19 and healthy donors. These cells were stained with FAM-YVAD-FLICA (**A**, **B**) or propidium iodide (PI) (**C**, **D**). Plasma samples were analyzed for IL-1β levels (**E**). Western blotting, histogram and graphs data are representative of nine critically ill patients and eight healthy donors. Data are presented as the mean ± SEM ^*^P < 0.05 versus healthy donors (HD).

To clinically validate our findings, we evaluated if monocytes obtained from critically ill patients with COVID-19 would also display signals of pyroptosis-like caspase-1-dependent cell death. We observed that monocytes from COVID-19 patients had increased caspase-1 activation (Fig. 5A, B) and significantly higher lytic death, by PI+ staining, when compared to monocytes from healthy donors (HD) (Fig. 5C, D). Corroborating with our in vitro data, we also detected higher levels of IL-1β in the plasma of critically ill patients, compared to HD (Fig. 5E). Therefore, the in vitro results from the previous sections stand on the shoulders of the clinical relevance of monocytes in patients with severe COVID-19.

## Discussion

COVID-19 has caused in over 100,000 deaths/month worldwide^21^ and represent the major public health crisis of the beginning of 21st century, leading to an unpredictable impact in global economics^25,26^. SARS-CoV-2 infection triggers an uncontrolled inflammatory response and marked leukopenia with consequent lung/respiratory dysfunction^27,28^. Similarly, to other respiratory viruses^29–31^, SARS-CoV-2 induces a cytokine storm, characterized by an uncontrolled inflammatory response mediated by monocytes/macrophages, when they should orchestrate the antiviral immune response^32^. In this work, we demonstrate that SARS-CoV-2 engages inflammasome, with subsequent caspase-1 activation, increase IL-1ß levels and GSDMD pore formation in human primary monocytes, pointing toward a pyroptotic cell death. This deleterious immune dysregulation loop triggered by SARS-CoV-2 may be impaired by ATV, glyburide and the blockage of the IL-1 receptor.

Our results demonstrate that SARS-CoV-2 leads to an intense cell death in human primary monocytes, observed by the increase in LDH release in infected cultures, as well as by the higher number of PI^+^ cells when compared to uninfected controls, both in experimental infection and in patients with severe COVID-19. Pyroptosis is an inflammatory and lytic programmed cell death triggered after inflammasome engagement^18–20^, it has been demonstrated that NLRP3 inflammasome activation in peripheral blood mononuclear cells (PBMCs) associated with severe COVID-19^21^. We confirmed that SARS-CoV-2 infection triggers NLRP3 inflammasome engagement in monocytes, because glyburide, a drug for treatment of type 2 diabetes which modulates ATP-dependent K^+^ channel^22^, could prevent virus-induced cell lysis. Next, we further demonstrated that downstream events could be prevented by early inhibition of virus replication, at protein translation levels, by ATV. Conversely, inhibition of viral RNA synthesis, by RBV did not affect cell death. Based on analogy with SARS-CoV from 2002, late viral proteins E, Orf3, and Orf8, which are synthetized after RNA synthesis, have been implicated in inflammasome activation^33^. Thus, it likely that other nonstructural proteins (nsp) from SARS-CoV-2 can affect intracellular K^+^ imbalance to trigger NLRP3. Among the possible SARS-CoV-2 proteins, nsp6 may affect K^+^ channels^34^.

Moreover, activation of caspase-1, increased production of IL-1ß and GSDMD pore was documented here in SARS-CoV-2-infected monocytes, points towards pyroptotic cell death These in vitro results are in accordance with the literature and with our finding described here that indicate the formation of inflammasomes in patients with severe COVID-19^35^. We also showed that the release of IL-1ß could be promoted by the activation of inflammasomes during the SARS-CoV-2 infection, because blockage of IL-1ß receptors reduced caspase-1 activation and cell death. These results corroborate with studies showing that the increase in IL-1ß production is associated with severe COVID-19^36–43^. Under our experimental conditions, the increase in IL-1ß precedes the unbalanced IL-6 release. These information should not be neglected when considering biopharmaceuticals to tackle cytokine storm in severe COVID-19.

To establish the clinical relevance of SARS-CoV-2-induced pyroptosis in monocytes^44,45^, we analyzed peripheral monocytes isolated from patients with severe COVID-19. We found that the cells from the patients displayed higher caspase-1 activation, when compared with monocytes isolated from HD. Recent clinical data reveal high levels of LDH and consistent leukopenia in critically ill COVID-19 patients^6,7,35,42–46^. Our data also demonstrate intense monocyte death in COVID-19 patients, as detected through flow cytometry analyzes. Altogether, these data suggest that the severity of COVID-19 may be associated with inflammasome activation in monocytes that results in large amounts of IL-1ß and generates an excessive inflammatory response, further characterized by high levels of IL-6 and TNF-α. Consistently, treatment inhibition of IL-1 receptor has been associated with clinical and inflammatory improvements in critically ill COVID-19 patients^42^. These results are in line with clinical case reports that demonstrate that monocytes/macrophages are key cells in the deleterious pro-inflammatory events that characterize the most serious cases of COVID-19^47–49^.

In this work, we originally describe that infection by SARS-CoV-2 can engage inflammasome activation and pyroptotic cell death, which may be related to the intense leukopenia and exacerbated inflammation seen in severe cases of the COVID-19. Since there is no specific therapy for this disease, our results point out that ATV has a promising therapeutic potential against SARS-CoV-2-induced cell death.

## Material and methods

### Reagents

ATV and RBV were received as donations from Instituto de Tecnologia de Fármacos (Farmanguinhos, Fiocruz). The antiviral LPV/ritonavir (4:1 proportion) was pruchased from AbbVie (Ludwingshafen, Germany). ELISA assays were purchased from R&D Bioscience. Lipopolysacchadides—LPS, adenosine triphosphate (ATP), the specific inhibitor of caspase-1 (AC-YVAD-CMK), pan-caspase inhibitor (ZVAD-FMK), RIPK1 (Necrostatin-1—Nec-1), IL-1 receptor (IL-1RA) and glyburide were all purchased from Sigma-Aldrich (St. Louis, MO, USA). All small molecule inhibitors were dissolved in 100 % dimethylsulfoxide (DMSO) and subsequently diluted at least 10^4^-fold in culture or reaction medium before each assay. The final DMSO concentrations showed no cytotoxicity. The materials for cell culture were purchased from Thermo Scientific Life Sciences (Grand Island, NY), unless otherwise mentioned.

### Cells and virus

African green monkey kidney (Vero, subtype E6) cells were cultured in DMEM high glucose supplemented with 10% fetal bovine serum (FBS; HyClone, Logan, Utah) and 100 U/mL penicillin, and 100 μg/mL streptomycin (P/S). Vero cells were incubated at 37 °C in 5% CO_2_ atmosphere.

Human primary monocytes were obtained through plastic adherence of PBMCs, which were obtained from buffy coat preparations of HDs by density gradient centrifugation (Ficoll–Paque, GE Healthcare). In brief, PBMCs (2.0 × 10^6^ cells) were plated onto 48-well plates (NalgeNunc) in RPMI-1640 without serum for 2–4 h; then, non-adherent cells were removed by washing and the remaining monocytes were maintained in DMEM with 5% human serum (HS; Millipore) and penicillin/streptomycin.

SARS-CoV-2 was isolated and expanded on Vero E6 cells from a nasopharyngeal swab of a confirmed case from Rio de Janeiro, Brazil. Experiments were performed after one passage in cell culture, when Vero E6 cells with DMEM plus 2% FBS in 150 cm^2^ flasks were incubated at 37 °C in 5% CO_2_ atmosphere. Cytopathic effect was observed daily and peaked 4–5 days after infection. All procedures related to virus culture were handled at biosafety level 3 (BSL3) multiuser facility, according to WHO guidelines. Virus titers were determined as the tissue culture infectious dose at 50% (TCID50/mL). Virus stocks were kept in −80 °C ultralow freezers. The virus strain was sequenced to confirm the virus identity and its complete genome is publicly deposited (GenBank accession No. MT710714).

### Yield-reduction assay

Human primary monocytes were infected with multiplicity of infection (MOI) of 0.01 at density of 2–8 × 10^5^ cells/well in 48-well culture plates, depending on the total cell number from each donor. After 1 h at 37 °C, cells were washed, and various concentrations of compounds were added in DMEM with 2% FBS. After 48 h, the supernatants were harvested and virus replication was quantified by real time RT-PCR and infectious titers by TCID50/mL.

### Virus titration

Monolayers of Vero cells (2 × 10^4^ cell/well) in 96-well culture plates were infected with log-based dilutions of the supernatants containing SARS-CoV-2 for 1 h at 37 °C. The cells were washed and fresh medium with 2% FBS was added. After 3–5 days, the cytopathic effects were scored in at least ten replicates per dilution by independent readers, who were blind with respect to source of the supernatant. Reed and Muench scoring method was employed to determine TCID50/mL.

### Flow cytometer analysis

For flow cytometry analysis, monocytes were diluted in labeling buffer (10^6^ cells/mL). Then, 100 µL of cell samples were marked with 5 µL of AnnexinV and PI for 15 min for cell death evaluation. Around 10,000 gated events were acquired using FACSCalibur and the analysis was performed using the CellQuest software. Monocytes were gated through cell size (foward light scatter) and granularity (side light scatter) analysis.

Human monocytes were stained for caspase‐1 activity with FAM‐YVAD‐FMK (fluorescent‐labeled inhibitor of caspase‐1 [FLICA] and FAM-FLICA Caspase-3/7 activity or HLA-DR APC.H7 or IgG APC.H7. Caspase-1 and caspase-3/7 activity was determined via flow cytometry (FACSCalibur) by detecting FLICA fluorescence and expression of HLA-DR as mean fluorescence intensity (MFI) value for each sample. Acquisition of data was set to count a total of 10,000 events, and the FLOWJO software package was used to analyze the data.

### Microscopic analysis

Human primary monocytes were plated on glass coverslips at density of 2–8 × 10^5^ cells/well in 48-well plates. Infection was performed for 2 h at 37 °C and then fresh medium with 2% FBS was added. After 24 h, the cells were washed with Binding buffer and stained with PI (0.5 µg/mL) for 5 min. Next, the cells were fixed with 3.7% formaldehyde for 30 min at room temperature. The nuclei were stained with DAPI (1 µg/mL) for 5 min and the coverslips were mounted using an antifade mounting medium (VECTASHIELD). Fluorescence was analyzed by fluorescence microscopy with an ×100 objective lens (Olympus, Tokyo, Japan).

### Measurements inflammatory mediators and cell death marker

The levels of IL-1ß, TNF-α, IL-6, IL-8, and LDH were quantified in the culture supernatants from infected and uninfected monocytes using ELISA kits, according to the manufacturer’s intructions (R&D System).

Extracellular LDH was quantified using Doles^®^ kit according to manufacturer’s instructions. In brief, cell culture supernatants were centrifuged at 5000 rpm for 1 min, to remove cellular debris, and then 25 μL were placed into 96-well plates and incubated with 5 μL of ferric alum and 100 μL of LDH substrate for 3 min at 37 °C. Nicotinamide adenine dinucleotide (oxidized form) was added followed by the addition of a stabilizing solution. After 10 min, the reaction was read in a spectrophotometer at 492 nm.

### Western blot assay

Cellular extracts of 1 × 10^6^ cells were homogenized in the RIPA lysis buffer in the presence of proteinase inhibitor cocktail (Roche), and the protein levels were measured by bicinchoninic acid assay protein assay kit. A total of 20 μg of protein was loaded onto a 10% sodium dodecyl sulfate polyacrylamide gel for separation by electrophoresis and the protein bands were then transferred to a polyvinylidene difluoride membranes (ImmobilonP-SQ, Millipore). The membranes were blocked with 5% albumin diluted in Tris-buffered saline containing 0.05% of Tween 20 for 2 h at room temperature and incubated with the specific primary antibodies (Cell Signaling Technology), to detect pro-caspase-1 and cleaved-caspase-1, after overnight incubation at 4 °C. After washing, membranes were incubated with secondary antibodies (IRDye^®^ 800CW Goat-anti-Mouse and IRDye^®^ 680LT Goat anti-Rabbit IgG Antibody, LI-COR, Lincoln) for 30 min at RT. The protein bands were visualized by digital fluorescence (Odyssey^®^ CLx Imaging System), and protein density was analyzed by the ImageJ software. All the data were normalized by β-actin expression quantification.

### Human subjects

We prospectively enrolled severe COVID19 RT-PCR-confirmed cases, as well as SARS-CoV-2-negative healthy controls. Blood samples were obtained from 12 patients with severe COVID-19 within 72 h from intensive care unit admission in two reference centers (Instituto Estadual do Cérebro Paulo Niemeyer and Hospital Copa Star, Rio de Janeiro, Brazil). Severe COVID-19 was defined as critically ill patients presenting viral pneumonia on computed tomography scan and in mechanical ventilation. All patients had SARSCoV-2 confirmed diagnostic through RT-PCR of nasal swab or tracheal aspirates. Peripheral vein blood was also collected from eight SARS-CoV-2-negative healthy control participants as tested by RT-PCR on the day of blood sampling. The characteristics of severe (*n* = 12), and control (*n* = 8) participants are presented in Table 1. Severe COVID-19 patients usually present older age and higher prevalence of comorbidities as obesity, cardiovascular diseases and diabetes as in previously reported patient cohorts. In the present study, the SARS-CoV-2-negative control group was designed to include subjects of older age and chronic non-communicable diseases, so it is matched with critically ill COVID-19 patients (Table 1). Patients with ARDS were managed with neuromuscular blockade and a protective ventilation strategy that included low tidal volume (6 mL/kg of predicted body weight) and limited driving pressure (less than 16 cm H_2_O) as well as optimal PEEP calculated based on the best lung compliance and PaO_2_/FiO_2_ ratio. In those with severe ARDS and PaO_2_/FiO_2_ ratio below 150 despite optimal ventilatory settings, prone position was initiated. Our management protocol included antithrombotic prophylaxis with enoxaparin 40–60 mg per day. Patients did not receive routine steroids, antivirals, or other anti-inflammatory or anti-platelet drugs. The SARS-CoV-2-negative control participants were not under anti-inflammatory or anti-platelet drugs for at least 2 weeks. All clinical information were prospectively collected using a standardized form—ISARIC/WHO Clinical Characterization Protocol for Severe Emerging Infections (CCPBR).

**Table 1:** Characteristics of COVID-19 patients and control subjects.

Characteristics^a^	Control (*n* = 8)	Covid-19 (*n* = 9)
Age, years	52.6 (47–60)	61 (33–79)
Sex, male	8 (100%)	6 (66.7%)
*Respiratory support*		
Oxygen supplementation	0 (0%)	3 (33.3%)
Mechanical ventilation	0 (0%)	6 (66.7%)
*SAPS 3*		59.25 (31–75)
PaO_2_/FiO_2_ ratio	–	210.75 (87–509.5)
Vasopressor	–	3 (33.3%)
Time from symptom onset to blood sample, days	–	10 (6–18)
*Status on Jun 30th*		
Dead	–	5 (55.55%)
Discharged	–	1 (11.1%)
Hospitalized	–	3 (33.3%)
*Comorbidities*		
Obesity	1 (11.1%)	1 (11.1%)
Hypertension	3 (33.3%)	3 (33.3%)
Diabetes	0 (0%)	0 (0%)
Cancer	0 (0%)	0 (0%)
Chronic heart disease^b^	0 (0%)	0 (0%)
*Presenting symptoms*		
Cough	0 (0%)	3 (33,3%)
Fever	0 (0%)	3 (33,3%)
Dyspnea	0 (0%)	4 (44,4%)
Headache	0 (0%)	0 (0%)
Anosmia	0 (0%)	0 (0%)
*Laboratory findings on admission*		
Lymphocyte count, cells/mm^3^	–	1355.8 (552–2564)
Platelet count, ×1000/mm^3^	–	169.6 (92–278)
Leukocytes	–	8036 (8.03–18,670)
C reactive protein, mg/L	–	14.78 (0.1–30.8)

^a^Numerical variables are represented as the median and the max range, and qualitative variables are represented as the number and the percentage.

^b^Coronary artery disease or congestive heart failure

### Ethics statement

Experimental procedures involving human cells from HDs were performed with samples obtained after written informed consent and were approved by the Institutional Review Board (IRB) of the Oswaldo Cruz Foundation/Fiocruz (Rio de Janeiro, RJ, Brazil) under the number 397-07. The National Review Board approved the study protocol (CONEP 30650420.4.1001.0008), and informed consent was obtained from all participants or patients’ representatives.

### Statistical analysis

The assays were performed in blinded way. They were performed by one professional, codified and read by another fellow. All experiments were carried out at least six independent times, including a minimum of two technical replicates in each assay. The equations to fit the best curve were generated based on *R*^2^ values ≥ 0.9. Student’s *t* test was used to access statistically significant *P* values < 0.05.

## Supplementary information

SUPPLEMENTARY FIGURES LEGENDS

FIGURE S1

FIGURE S2

FIGURE S3
