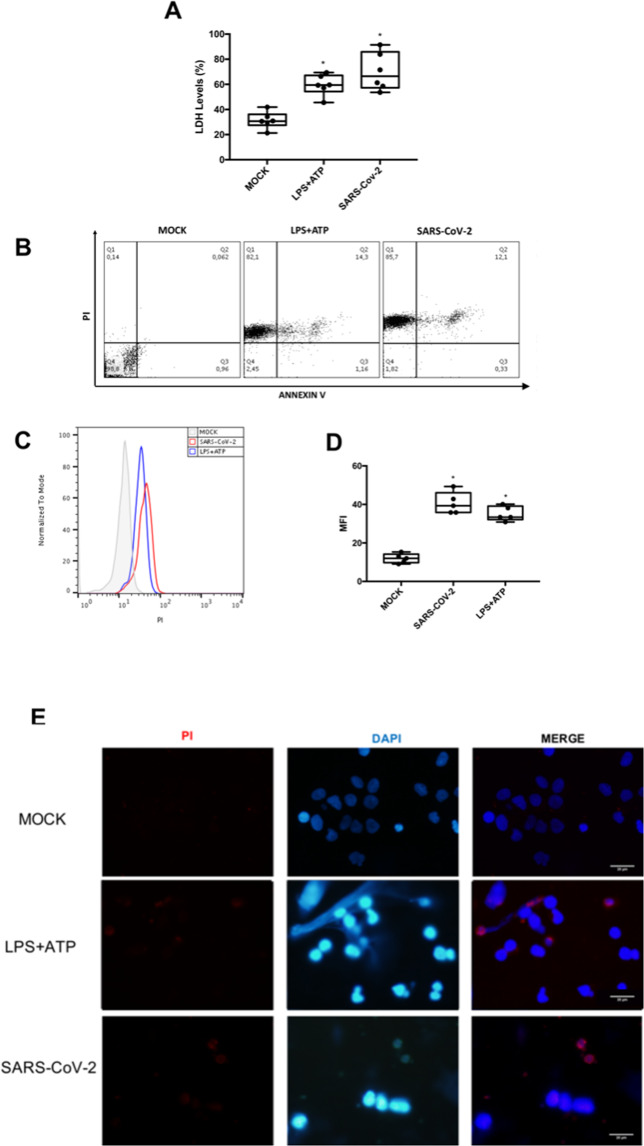# Correction: SARS-CoV-2 engages inflammasome and pyroptosis in human primary monocytes

**DOI:** 10.1038/s41420-021-00477-1

**Published:** 2021-05-19

**Authors:** André C. Ferreira, Vinicius Cardoso Soares, Isaclaudia G. de Azevedo-Quintanilha, Suelen da Silva Gomes Dias, Natalia Fintelman-Rodrigues, Carolina Q. Sacramento, Mayara Mattos, Caroline S. de Freitas, Jairo R. Temerozo, Lívia Teixeira, Eugenio Damaceno Hottz, Ester A. Barreto, Camila R. R. Pão, Lohanna Palhinha, Milene Miranda, Dumith Chequer Bou-Habib, Fernando A. Bozza, Patrícia T. Bozza, Thiago Moreno L. Souza

**Affiliations:** 1 Laboratório de Imunofarmacologia, Instituto Oswaldo Cruz (IOC), Fundação Oswaldo Cruz (Fiocruz), Rio de Janeiro, RJ Brazil; 2 Laboratório de Pesquisa Pré-clínica—Universidade Iguaçu - UNIG, Nova Iguaçu, RJ Brazil; 3 National Institute for Science and Technology on Innovation in Diseases of Neglected Populations (INCT/IDPN), Center for Technological Development in Health (CDTS), Fiocruz, Rio de Janeiro, RJ Brazil; 4 Program of Immunology and Inflammation, Federal University of Rio de Janeiro, UFRJ, Rio de Janeiro, RJ Brazil; 5 Laboratório de Pesquisas sobre o Timo, IOC, Fiocruz, Rio de Janeiro, RJ Brazil; 6 National Institute for Science and Technology on Neuroimmunomodulation, Oswaldo Cruz Institute, Fiocruz, Rio de Janeiro, RJ Brazil; 7 Laboratório de Imunotrombose, Departamento de Bioquímica, Universidade Federal de Juiz de Fora, Juiz de Fora, MG Brazil; 8 Laboratório de Vírus Respiratório e do Sarampo, IOC, Fiocruz, Rio de Janeiro, RJ Brazil; 9 Instituto Nacional de Infectologia Evandro Chagas, Fiocruz, Rio de Janeiro, RJ Brazil; 10 Instituto D’or de Pesquisa e Ensino, Rio de Janeiro, RJ Brazil

**Keywords:** Viral infection, Inflammasome

Correction to: *Cell Death Discovery*

10.1038/s41420-021-00428-w published online 1 March 2021

The original version of this article unfortunately contained a mistake. The authors noticed an accidental duplication of Fig. 1E. The correct figure can be found below. The original article has been corrected.