# Supplementary material for: SARS-CoV-2 engages inflammasome and pyroptosis in human primary monocytes
Source: Cell Death Discov. 2021 Mar 1;7:43. doi: 10.1038/s41420-021-00428-w (PMC7919254; doi:10.1038/s41420-021-00428-w)
Supplement: Supplementary file 1 — SUPPLEMENTARY FIGURES LEGENDS [file 41420_2021_428_MOESM1_ESM.docx]

Supplementary Material Legends

SARS-CoV-2 engages inflammasome and pyroptosis in human primary monocytes

**Figure S1. SARS-CoV-2-induced monocyte death and caspase-1 activation depends on IL-1β.** Human monocytes were pretreated with IL-1β receptor inhibitor (IL-1RA; 1 μM) for 1 h and infected with SARS-CoV-2 (MOI 0.1) for 24 h. (A) Cell viability was assessed through the measurement of LDH levels in the supernatant. (B) monocytes were stained with FAM-YVAD-FLICA to determine the caspase-1 activity. (C) Cell culture supernatants also were collected for the measurement of the levels of IL-1β. Data are presented as the mean ± SEM # P < 0.05 versus infected and untreated group; * P < 0.05 versus control group (MOCK-infected). Graphs are representative of four independent experiments.

**Figure S2. SARS-CoV-2-induced IL-8 enhancement in monocytes is independent of caspase-1 and IL-1 β.** Human monocytes were treated with caspase-1 inhibitor (AC- YVAD-CMK; 1 μM), pan-caspase inhibitor (ZVAD-FMK; 10 μM), or RIPK inhibitor (Nec-1; 25 μM) or with inhibitor of IL-1ß receptor (IL-1RA; 1 μM). Monocytes were stimulated with LPS (500ng/mL) for 23 h and after this time were stimulated with ATP (2 mM) for 1 h as a positive control. Cell culture supernatants were collected for the measurement of the levels of IL-8. Graph data is representative of six independent experiments. Data are presented as the mean ± SEM # P < 0.05 versus infected and untreated group.

**Figure 3. Evaluation of capase-1 and -3/7 activity upon treatment with generic antivirals in LPS+ATP-induced monocytes.** Human monocytes were stimulated with LPS (500ng/mL) for 23 h and, after this time, with ATP (2 mM) for 1 h. These cells were treated or with atazanavir (ATV; 10 μM), lopinavir (10 μM) or ribavirin (10 μM) for 24 h. Monocytes were stained with FAM-YVAD-FLICA (A) or FAM-FLICA (B) to determine the caspase-1 and caspase-3/7 activity, respectively. Histograms are representative of six independent experiments.
