# Supplementary figures and images for: SARS-CoV-2 engages inflammasome and pyroptosis in human primary monocytes
Source: Cell Death Discov. 2021 Mar 1;7:43. doi: 10.1038/s41420-021-00428-w (PMC7919254; doi:10.1038/s41420-021-00428-w)

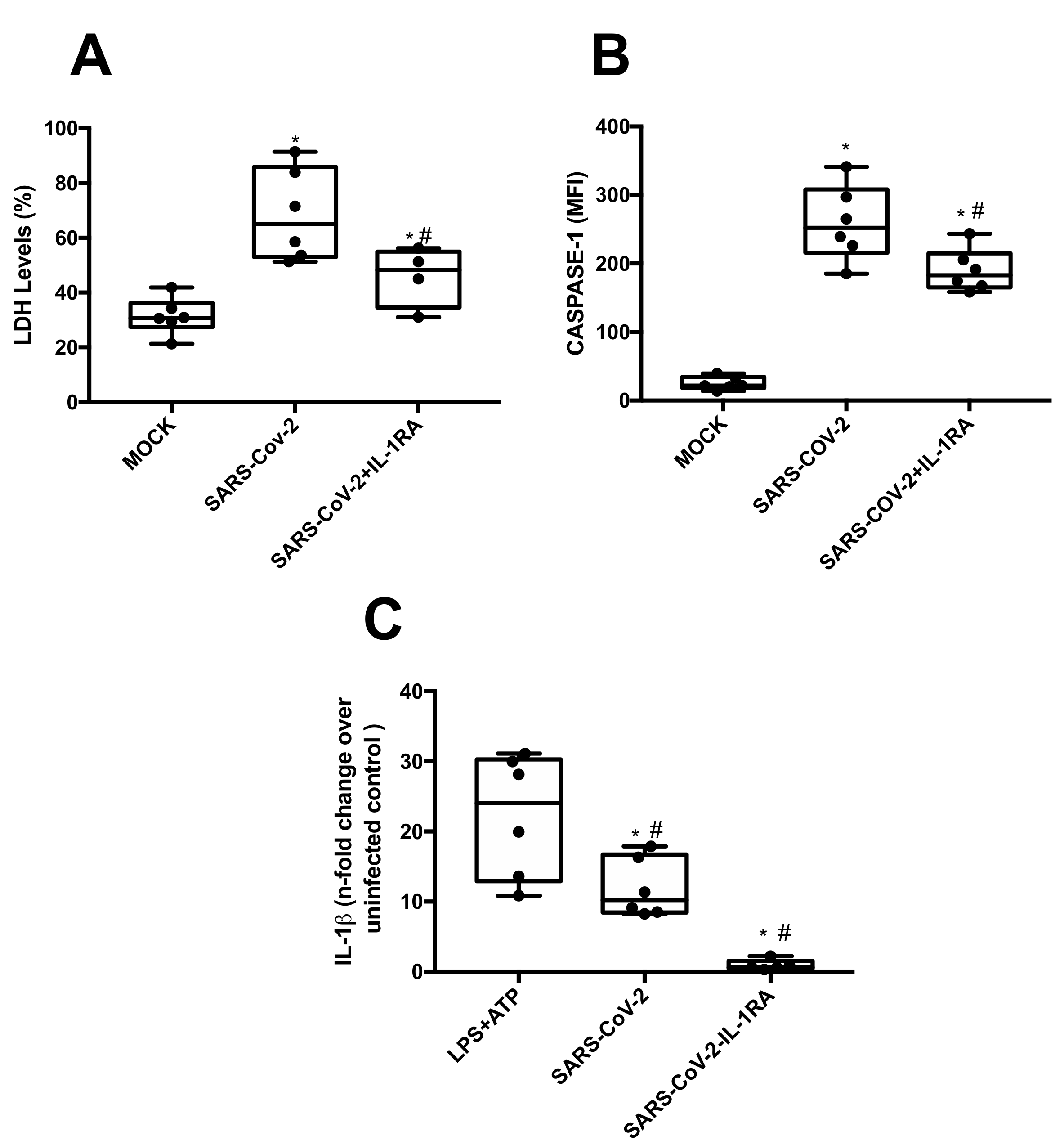

Supplement: Supplementary file 2 — FIGURE S1 [file 41420_2021_428_MOESM2_ESM.tif]

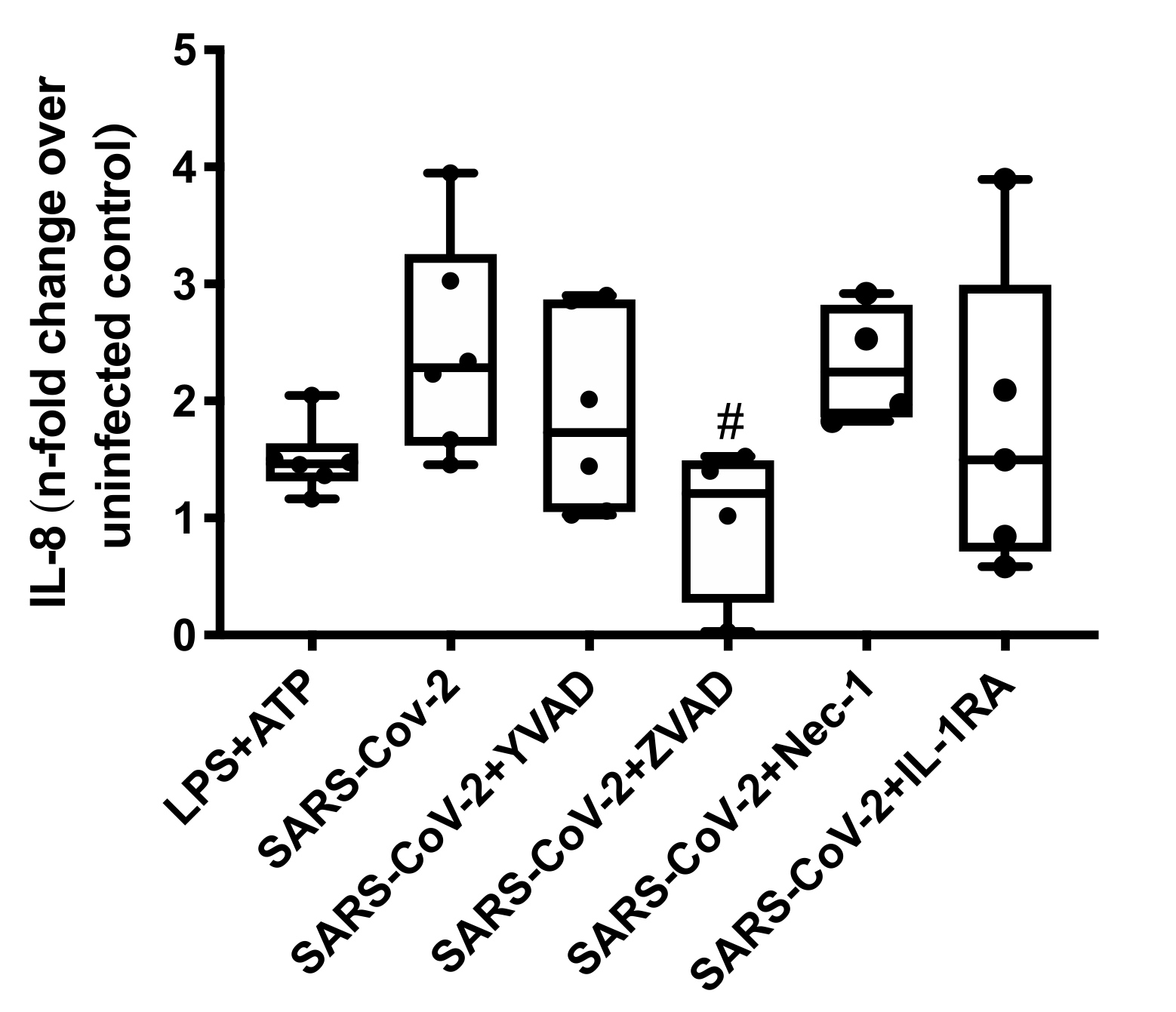

Supplement: Supplementary file 3 — FIGURE S2 [file 41420_2021_428_MOESM3_ESM.tif]

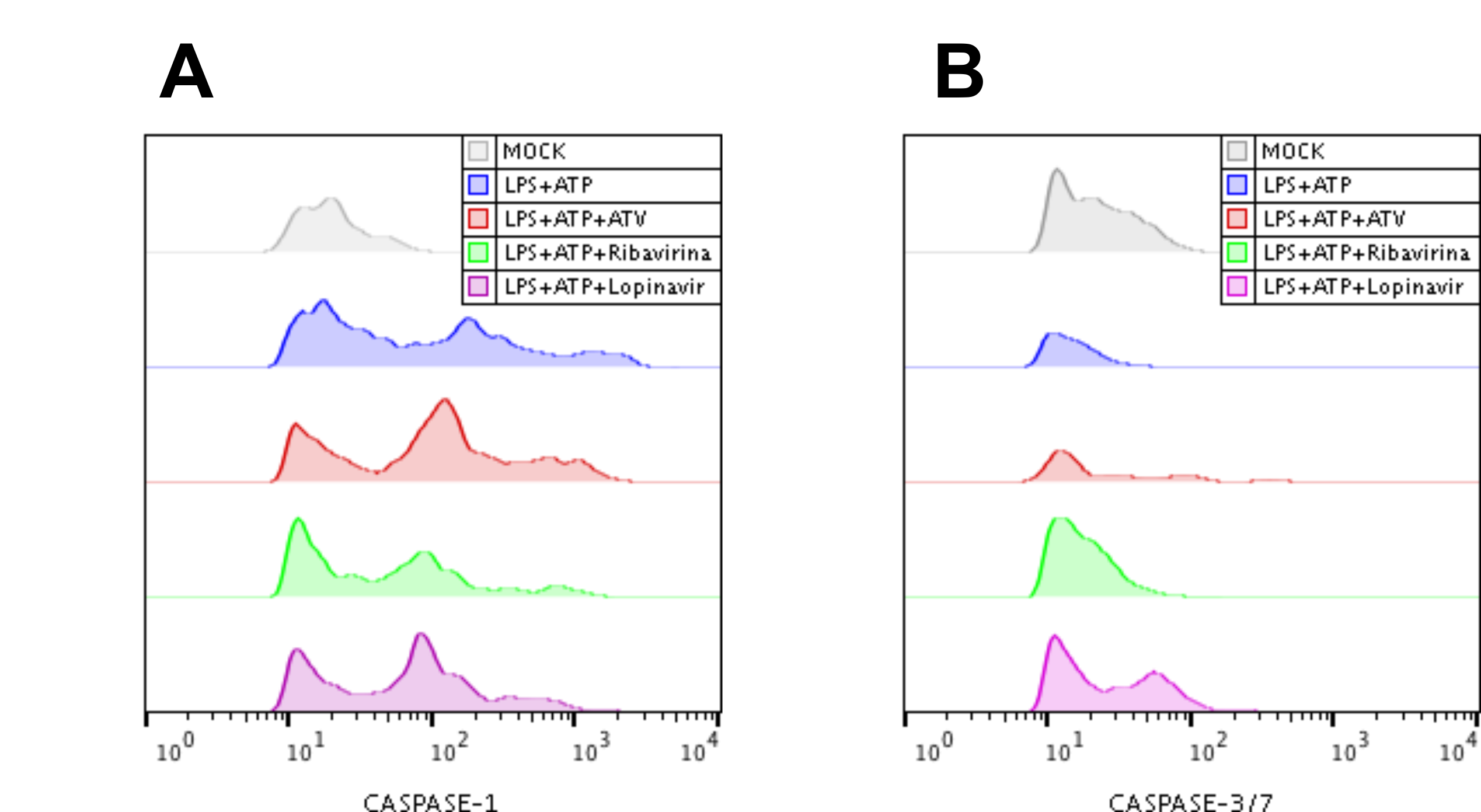

Supplement: Supplementary file 4 — FIGURE S3 [file 41420_2021_428_MOESM4_ESM.tif]
